# Hospital Progress in Paris.—III

**Published:** 1905-06-03

**Authors:** 


### HOSPITAL ADMINISTRATION.

### CONSTRUCTION AND ECONOMICS.

### HOSPITAL PROGRESS IN PARIS.—III.

By the Author of "Hospital Expenditure: The Commissariat."

*Concluded from page 143.*

It has been shown in preceding articles that the central system of administration, regarded under its highest form of development in Paris, appears rather to retard than to advance reforms, and proves, in so far as general efficiency is concerned, an unwieldy engine. And its defects can be traced quite apart from those inherent in municipal management. From the worst conceivable condition, with but slight encouragement from the medical profession, and with none from the public, who are content with the privilege of grumbling under any and every circumstance, the Poor-law infirmaries of London and many provincial towns have one by one risen to the rank of perhaps the best institutions of their kind in the world. And the secret of the difference between their condition to-day and that of some Parisian "hospices" is to be sought in the word "freedom." It is by no means true that London is more interested in the condition of the aged and chronically infirm than Paris. The contrary might almost be asserted, for in Paris the most anxious care is taken to uphold "the rights of the poor" and to deliver from any stigma those who are indebted to "public assistance." But the resistless chain of uniformity and centralisation has perforce stifled the natural growth of philanthropy, which, starting experimentally in one locality, is first admired, then imitated in another, and then handed on in an improved form. To convince every official on a central board of the practicability of some desirable step before it has even been tried is a task to daunt enthusiasm, even if enthusiasm were not always itself suspect in high quarters.

But has centralisation altogether failed? We believe that it has obstructed progress during the transition period from the old to the new through which medicine and surgery have recently passed, perhaps are still passing.

But it does not follow that in all particulars the system is impracticable. It has produced at least one brilliant success worthy of consideration by the strongest upholders of the "free and independent institution." We allude to the central supply system under which bread, meat, milk, stores of all kinds, coke and coal, household requisites, linen, bedding, clothing, furniture, drugs, etc., etc., are all furnished at the lowest possible cost to the institutions of the "Assistance Publique." These institutions make up a total of 16,240 hospital beds, and afford besides hospice accommodation for 13,182 aged, chronic, and mentally afflicted cases. Counting supplementary beds, there are about 30,000 persons to be fed, lodged and clothed from the central stores, in addition to the staff, which cannot be less than 5,000 more. The establishments charged with the distribution of stores are six in number: pharmacy, bakery, meat store, wine store, perishable provision stall (at the market) and central stores for everything not provided elsewhere. In addition, there is a central laundry. An excellent description of the system will be found in "Burdett's Hospitals and Charities," Vol. III. The service has been improved and added to since 1887, but remains substantially the same. An effort should be made to see at least one or two of these admirable establishments when visiting Paris, and it is worth noting that the visit should be paid before 6 a.m., if it is desired to see them in working order at full pressure.

The advantages of centralisation (or shall it be called co-operation?) in such an undertaking are at once perceptible.

First, every institution is relieved from the onus of examining contracts, studying market prices and supervising consignments of goods. All this is expert work. It is outside the talents which go to make up a good director or superintendent, and must be delegated by him to subordinates unless an undue proportion of his time is to be absorbed in such details. At the Central Stores highly trained officials give their whole time to the business. They receive tenders direct from the manufacturers and producers, and can bring expert knowledge founded on long experience to bear on the question of prices and quality. A competent staff is always at work weighing, tasting, measuring, testing in every way the consignment of goods delivered, and by this means every article not up to the required standard is instantly detected and returned. Every bale of calico is stretched and examined for flaws; every cask of wine must pass the taster; the wool for the mattresses comes under a trained eye quick to detect if the quality answers to the sample selected; the bullock for the slaughter must give account of his health; the corn, ground at the central mill, passes through the fingers of a miller who can place its locality by its appearance and estimate its value to a minute fraction. It is not possible that any hospital steward, however able he may be, can command the varied and unerring skill of such a staff.

Secondly, the magnitude of the operations undoubtedly favours a reduction in price. There is keen competition for the custom of the hospital service, for it pays well to cut profits to a minimum in the case of large transactions supported by prompt payments. The dangers attendant upon low contracts are guarded against by the system of expert inspection we have described, and the gain to the public administration is such as guardians of the poor in London would do well to consider.

Thirdly, the system admits of securing able men above suspicion of collusion with those who supply the goods. The extent to which the domestic staff and lower officials in institutions play into the hands of contractors is a subject which it offends many sensibilities to mention. But so long as an unfortunate tone of morality permits persons strictly honourable in all other dealings to look leniently for a consideration on the shortcomings of the tradesmen, a very dangerous source of leakage will continue to exist in institution finance. The only way out of the difficulty is to attract men to this service of a higher calibre, and it is only through co-operation that the transactions become of sufficient importance to justify the appointment of highly salaried officers. It is by co-operation alone that those who are responsible for the use and distribution of provisions and other articles can be entirely dissociated from contact with the salesman.

Fourthly, the central system makes it possible and lucrative to undertake much which separate institutions cannot cope with. We have recently had evidence in the pages of The Hospital that it does not pay any but the largest hospitals to make their own preparations in a properly-equipped laboratory. The consumption is not sufficient to keep even a small staff constantly employed, and salaries overbalance the admittedly large gain over the price of these costly articles when bought wholesale. A visit to the Magasin Central will find the courteous director ready to explain how many processes deemed altogether foreign to hospital endeavour can be carried through at great saving of time and money. We confess that the branch of the work which pleased us most was that in which bandages of every kind were being prepared for subsequent sterilisation at the hospitals by groups of the old and infirm from the municipal almshouses. These old people, seated in well-warmed workrooms, with hours suited to their infirmities, were taking obvious pleasure in their employment, by which they were enabled to earn a modest pay and procure the little luxuries which mean so much in almshouse life. Nearly all the needlework for the vast establishments of the Assistance Publique is done by the poor of Paris, the widows, the wives of those who are out of work, the friendless and forlorn. Of these 400 are employed in the workrooms and 1,200 work in their own homes.

Might not the Poor-law authorities in London find a lesson in the relief of distress from an inspection of this admirably organised service?

